# Guiding Oligodendrocyte Progenitor Cell Maturation Using Electrospun Fiber Cues in a 3D Hyaluronic Acid Hydrogel Culture System

**DOI:** 10.1021/acsbiomaterials.4c01455

**Published:** 2024-12-20

**Authors:** Rachel A. Mazur, Kyle J. Lampe

**Affiliations:** Department of Chemical Engineering, University of Virginia, Charlottesville, Virginia 22903-1738 United States

**Keywords:** oligodendrocyte, oligodendrocyte progenitor cells, electrospinning, tissue engineering, biomaterials, hydrogels

## Abstract

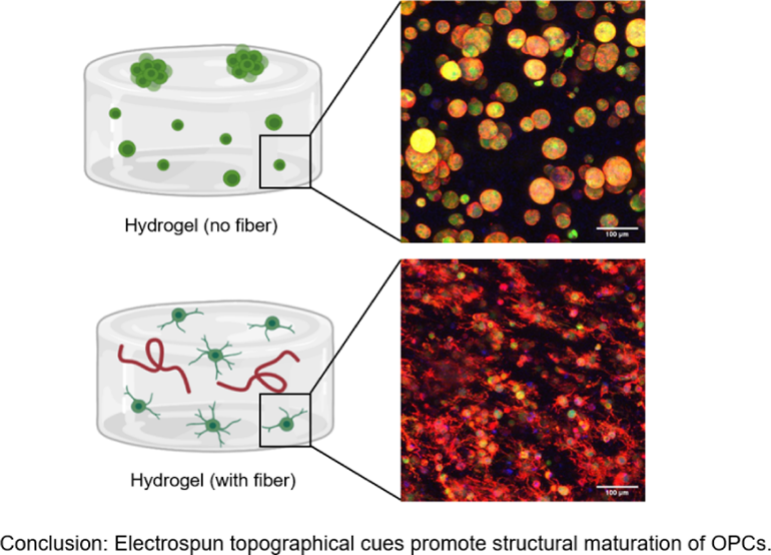

The current lack of therapeutic approaches to demyelinating disorders and injuries stems from a lack of knowledge surrounding the underlying mechanisms of myelination. This knowledge gap motivates the development of effective models to study the role of environmental cues in oligodendrocyte progenitor cell (OPC) maturation. Such models should focus on determining, which factors influence OPCs to proliferate and differentiate into mature myelinating oligodendrocytes (OLs). Here, we introduce a hyaluronic acid (HA) hydrogel system composed of cross-linked HA containing encapsulated HA fibers with swollen diameters similar to mature axons (2.7 ± 0.2 μm). We tuned hydrogel storage moduli to simulate native brain tissue (200–2000 Pa) and studied the effects of fiber presence on OPC proliferation, metabolic activity, protein deposition, and morphological changes in gels of intermediate storage modulus (800 ± 0.3 Pa). OPCs in fiber-containing gels at culture days 4 and 7 exhibited a significantly greater number of process extensions, a morphological change associated with differentiation. By contrast, OPCs in fiber-free control gels maintained more proliferative phenotypes with 2.2-fold higher proliferation at culture day 7 and 1.8-fold higher metabolic activity at culture days 4 and 7. Fibers were also found to influence extracellular matrix (ECM) deposition and distribution, with more, and more distributed, nascent ECM deposition occurring in the fiber-containing gels. Overall, these data indicate that inclusion of appropriately sized HA fibers provides topographical cues, which guide OPCs toward differentiation. This HA hydrogel/fiber system is a promising *in vitro* scheme, providing valuable insight into the underlying mechanisms of differentiation and myelination.

## 1 Introduction and Background

In healthy central nervous system (CNS) tissue, neuronal axons are protected by a lipid-rich myelin sheath which increases the specificity and efficiency of electrical signal conduction.^1^ Demyelination occurs when a CNS injury or disorder causes degradation of the myelin sheath. Therapeutic strategies for minimizing demyelination and promoting remyelination are essential for addressing demyelinating disorders and injuries, which collectively affect over 5 million people throughout the world.^2,3^ Multiple sclerosis (MS), the most common demyelinating disorder, is estimated to affect 2.5 million individuals worldwide.^3^ Central nervous system injuries such as brain or spinal cord trauma can also result in demyelination.^4^ While some symptoms of demyelinating disorders and injuries can be managed, there is currently no treatment capable of reversing the underlying damage caused by demyelination.^5,6^

As oligodendrocytes (OLs) mature, they extend multiple processes which wrap around axons and compact to form the final myelin sheath.^7^ Demyelination can be initiated through direct physical or chemical insult to the oligodendrocyte (primary demyelination), or as a result of axonal loss (secondary demyelination).^8^ Demyelination leads to many neurological difficulties, including decreased cognitive function and impaired coordination. Remyelination can occur in vivo to some extent, as new oligodendrocyte progenitor cells (OPCs) migrate to the affected area, proliferate locally, and differentiate and mature into myelinating oligodendrocytes. However, the resulting sheaths are typically shorter and thinner than normal.^9^ Additionally, this process often fails due to inadequate migration of OPCs to the injury site (recruitment failure) or an inability of recruited OPCs to differentiate and mature into oligodendrocytes (differentiation failure).^9^ In order to engineer potential therapeutics, the underlying factors which govern myelination and remyelination must first be determined. Unfortunately, a knowledge gap persists in this area since the specific factors which influence OPC/OL development are under-studied.^10^

To further understand the mechanisms behind myelination, demyelination, and recovery, it is vital to identify specific factors capable of guiding neural stem cells (NSCs) or OPCs into mature myelinating oligodendrocytes. Of the studies which do exist, the vast majority have been conducted *in vivo.*(11,12) The existing *in vivo* studies provide insight into the roles of OPC/OL in demyelination and recovery, but rarely permit independent examination of key factors of interest. Furthermore, due to the presence of confounding factors in *in vivo* developmental cascades, it is often difficult to determine the precise impact of individual factors on OPC/OL growth and development.

In contrast to *in vivo* animal models, *in vitro* biomaterial systems are an emerging platform for studying responses to individual environmental cues at the cellular level. *In vitro* neural tissue models simulate important properties of the native extracellular matrix (ECM) but allow for precise and tunable control of model parameters not possible in vivo. Some examples of parameters with the potential to impact cell growth and differentiation include matrix stiffness, OPC/neuron coculture, topographical cues, integrin-ligand interactions, drug or growth factor delivery, and/or matrix degradation.^13−21^ As one example, storage modulus within the native brain tissue range (*G*′ = 200–2000 Pa) promotes cell viability and differentiation.^22,23^ Tuning these parameters within an engineered *in vitro* model permits investigation into the unique effects of individual factors while simultaneously eliminating potential confounding variables.

Hydrogel platforms are a promising choice for *in vitro* culture since they are biocompatible, tunable, and facilitate cell behaviors such as migration, proliferation, and differentiation. Previous work has demonstrated that OPCs and their parent cells (NSCs) can be cultured in 3D hydrogels of various types, including polyethylene glycol (PEG), chitosan, and collagen.^16,17,23,24^ Hyaluronic acid (HA) hydrogels are especially relevant for CNS applications, since HA is a key component of brain extracellular matrix (ECM), particularly within the perineural net.^25^ Norbornene-functionalized hyaluronic acid (NorHA) gels can be covalently cross-linked under UV light to reliably form gels within the range of native brain tissue.^22^ Our group has previously demonstrated OPC viability and growth in this system.^22^ This system also facilitates detection of nascent ECM by the encapsulated cells. Encapsulated cells are known to remodel their local microenvironment and deposit ECM proteins within HA and other hydrogel systems.^26^*In vivo,* OPCs have been shown to secrete ECM proteins such as laminin and fibronectin.^27,28^ However, the amount, pattern, and composition of OPC protein deposition remains undetermined. Our hydrogel system can be used to elucidate these factors and to determine how protein deposition by OPCs varies in response to fiber cues.

Topographical cues, such as electrospun fibers, are another matrix factor that influences OPC differentiation and OL myelination. When seeded onto electrospun polystyrene fibers with diameters similar to in vivo myelinated axons (0.3–4 μm), some OPCs differentiate and wrap the fibers, particularly for fibers of larger diameters (>0.4 μm).^29,30^ Fiber systems composed of other polymeric materials, including poly(trimethylene carbonate-*co*-ε-caprolactone) (P(TMC-CL)) and graphene oxide and laminin-coated polycaprolactone (PCL), also increased NSC differentiation into oligodendrocytes.^31,32^ Hydrogel-fiber composite materials have been explored for non-neural cell types in various contexts, including guiding angiogenesis or cardiomyocyte alignment.^33,34^ However, to the best of our knowledge, the effects of electrospun fiber topography on OPCs have only been conducted in 2D with stiff polymeric materials. 3D cultures containing other forms of topography, such as ablated microchannels and interfaces between bicontinuous hydrogel systems, have shown that topographical cues guide cell migration in a 3D context.^35,36^ Our novel culture system allows for independent tuning of factors such as bulk gel stiffness, fiber size, and fiber concentration. This independent control over individual parameters allows precise examination of the role of micron-scale hydrogel fiber topography in guiding cell behaviors, such as proliferation and differentiation, in a 3D context.

In this work, we use electrospun methacrylated hyaluronic acid (MeHA) fibers with swollen diameters representative of native axons (2.7 ± 0.2 μm) to simulate the micron-scale structural cues of native axons.^37^ Following fiber encapsulation in NorHA gels, nanoindentation and rheology were performed to test the effects of fiber inclusion on both the local and bulk gel moduli, respectively. We directly compared green fluorescent protein positive (GFP+) mosaic analysis with double markers (MADM) OPC cultures in fiber-containing hydrogels to fiber-free control gels over a seven-day period via immunostaining and examination of cell morphology. We performed an EdU-based proliferation assay and an ATP/DNA assay to assess how fiber presence impacted cell proliferation and metabolic activity, respectively. Finally, we conducted immunostaining assays to quantify how OPCs deposited laminin and fibronectin to remodel their local environments within the hydrogel.

## 2 Materials and Methods

### 2.1 Flask Preparation and Cell Culture

Sterile T75 (VWR) tissue culture flasks were coated with 5 mL of a 10 μg/mL polyornithine solution and incubated at 37 °C for a minimum of 4 h and rinsed with Dulbecco’s Phosphate-Buffered Saline (DPBS). MADM OPCs were a generous gift from Prof. Hui Zong’s lab.^38^ All cells were cultured in OPC proliferation media throughout all maintenance and experiments. Proliferation media consisted of Dulbecco’s Modified Eagle Medium (DMEM) with 4.5 g/L d-glucose, 4 mM l-glutamine, and 110 mg/L sodium pyruvate (Invitrogen), supplemented with N2 supplement (Invitrogen), B27 supplement (Invitrogen), and 1% penicillin-streptomycin (Invitrogen). Cells were seeded onto flasks at a seeding density of 1 × 10^4^ cells/cm^2^. Media was changed every 2 days, and cells were passaged at 80–90% confluence. During cell passaging, cells were detached using a solution of 0.125% (0.5×) trypsin, followed by rinsing the flask with 10 mL DPBS. The resulting cell solution was centrifuged at 1000 rpm for 5 min, after which the supernatant was aspirated. Cells were resuspended in 300 μL of PBS plus 4.5 g/L d-glucose (PBSG) for cell counting.

### 2.2 NorHA Synthesis

Two different methods were employed for the synthesis of the product, as a new and improved protocol (Method 2) was published and adopted during the course of our research. All evidence indicates that the resulting product is fundamentally identical. For further details, please refer to the accompanying NMR spectra (Figures S1 and S2).

Method 1: norbornene-functionalized hyaluronic acid (NorHA) was synthesized similar to previously published work.^39,40^ Briefly, hyaluronic acid *tert*-butyl ammonium salt (HA-TBA) was synthesized by allowing sodium hyaluronate (Lifecore, 74 kDa) to undergo proton exchange with Dowex 50W resin, filtering, titrating with TBA–OH to pH 7.05, and lyophilizing. HA-TBA was brought to room temperature and added to a round-bottom flask equipped with a stir bar. To achieve a 20–30% functionalization efficiency, 0.3 stoichiometric equivalents of 5-norbornene-2-methylamine were added and the flask was purged with nitrogen for 5 min to ensure anhydrous reaction conditions. Anhydrous dimethyl sulfoxide (DMSO) was then added by cannulation (0.5 mL DMSO per 0.1 g HA-TBA) and mixed until the HA-TBA was completely dissolved. 0.3 stoichiometric equivalents of benzotriazole-1-yl-oxy-tris(dimethylamino)-phosphonium hexafluorophosphate (BOP) were added via cannulation and the reaction was allowed to proceed for 2 h at room temperature. The reaction was then quenched with 10 mL of deionized water and dialyzed via a 6–8 kDa molecular weight cutoff tubing against dionized water for 5 days, changing the water twice daily. Products were vacuum filtered and dialyzed for an additional 5 days before lyophilizing.

Method 2: norbornene-functionalized hyaluronic acid (NorHA) was also synthesized using another method.^41^ 4-(4,6-Dimethoxy[1,3,5]triazin-2-yl)-4-methylmorpholinium chloride (DMTMM) was employed as a coupling agent. Hyaluronic acid (74 kDa, LifeCore) was dissolved at 25 mg/mL in 40 mL of 1 mM 2-(*N*-morpholino)ethanesulfonic acid (MES) buffer and pH was adjusted to 5.5 with 10 M NaOH. DMTMM coupling agent (VWR) was added to a concentration of 31 mg/mL and mixed until the DMTMM was completely dissolved (about 10 min). A volume of 0.271 mL of 5-norbornene-2-methylamine (TCI America) was added to achieve a 20–30% functionalization efficiency, and the mixture was stirred overnight at room temperature. The reaction was precipitated into 250 mL of cold 95% ethanol. Precipitate was collected by vacuum filtration and redissolved in 2 M brine solution, followed by transferring to presoaked dialysis tubing with a 6–8 kDa molecular weight cutoff. Dialysis was conducted against DI water for 24 h, followed by brine solution for 24 h, changing the water twice daily. A final round of dialysis was conducted against DI water for 24 h, followed by lyophilizing.

Purity and degree of norbornene functionalization were determined with ^1^H NMR (Varian Inova 500 MHz) using D_2_O as a solvent (Figures S1 and S2). Lyophilized NorHA products were stored under nitrogen at 4 °C.

### 2.3 MeHA Synthesis

Methacrylated hyaluronic acid (MeHA) was synthesized according to previously established protocols.^42,43^ Briefly, methacrylic anhydride (MA) was added dropwise to research grade sodium hyaluronate (Lifecore Biomedical, 74 kDa) at 1% w/v and stirred until homogeneous. The solution was kept on ice and the pH was maintained between 8 and 9 with 5 N NaOH. MA was added in a 1:1 molar ratio to HA with 12.3× molar excess. After all methacrylic anhydride was added, the solution was continually stirred overnight. The solution was then dialyzed (molecular weight cutoff 6–8 kDa) for 5 days against deionized water. Macromer solution was frozen overnight at −80 °C and lyophilized for 3 days. Purity and degree of methacrylate functionalization were determined with ^1^H NMR (Varian Inova 500 MHz) using D_2_O as a solvent (Figure S3). The final MeHA product was stored under nitrogen at 4 °C.

### 2.4 Electrospinning and Fiber Characterization

Electrospinning parameters were selected based on previously established protocols.^44,45^ Briefly, electrospinning solution was prepared in 2 mL batches containing 2 wt % MeHA, 3 wt % 9 kDa poly(ethylene oxide) (Sigma), 0.05 wt % Irgacure 2959 photoinitiator (I2959, Sigma) and 0.415 mg/mL methacrylated rhodamine B (Sigma) dissolved in deionized water. Solution was protected from light and allowed to dissolve in a 5 mL Cadence syringe for 24–48 h with stirring. Fibers were then spun using a Spraybase electrospinning machine at 21% relative humidity with a 21 cm collection distance, 8–11 kV voltage, 20-gauge needle and 0.4 mL/h flow rate. Dry fibers were collected on a sheet of aluminum foil and stored under nitrogen until cross-linking. Cross-linking was conducted for 15 min using an Omnicure UV light (365 nm) set to an intensity of 14 mW/cm^2^. Dry electrospun fibers were imaged using SEM with a voltage of 1.0 kV and working distance of 21 cm. Four images of different sites were sampled at 5,000× and 10,000× magnifications. Fiber diameters were determined using the length measuring tool in FIJI ImageJ (taking scale bar as a reference).^46^

Dry fibers were swollen overnight in deionized water and processed by repeatedly passing through an 18-gauge needle, followed by a 21-gauge needle similar to previously established protocols.^45^ This swollen fiber solution was used for subsequent hydrogel encapsulation. Swollen fiber solution (5 wt % in PBS) was applied to a microscope slide and imaged using a Zeiss inverted fluorescent microscope. Fluorescent signal from the tagged rhodamine B was imaged between 2.5 and 10× at multiple sites. Fiber diameters were determined using the length measuring tool in FIJI ImageJ, using the scale bar as a reference.^46^ Images of swollen fibers encapsulated in NorHA gels are shown in Figure S7.

### 2.5 Rheology

NorHA macromer solution was created by dissolving 1–2 wt % NorHA macromer, 0.0328 wt % lithium phenyl-2,4,6-trimethylbenzoylphosphinate (LAP) photoinitiator, and dithiothreitol (DTT, Sigma) at a 0.312 thiol-norbornene ratio in PBS. For fiber-containing conditions, electrospun fibers were also included at 1 wt % (based on dry mass). 75 μL aliquots were pipetted onto a UV-configured plate of an Anton Paar MCR 302 rheometer. A 25 mm diameter smooth cone and plate with a 0.505° angle was used to perform a time sweep. Gels were cured in situ under conditions previously determined to be within the LVE range (0.1% oscillatory strain and 10 rad/s oscillation frequency), and 4 mW/cm^2^ UV light intensity (365 nm). Samples were exposed to oscillatory motion in the absence of UV light for 30s at the start of each test. UV light exposure was introduced underneath the plate for 2 min, after which UV light was turned off and oscillatory motion continued for an additional 60s. Plateau storage modulus was calculated by averaging over the final 30s.

### 2.6 3D Cell Encapsulation and Hydrogel Culture

NorHA macromer solution was created by dissolving 1–2 wt % NorHA macromer, 0.0328 wt % LAP and dithiothreitol (DTT) at an 0.312 thiol-norbornene ratio in PBS. For fiber-containing conditions, gel precursor solution also contained swollen electrospun fiber solution at 1 wt % (based on preswollen fiber dry mass). OPCs were combined with precursor solution to a final concentration of 0.5 × 10^7^ cells/mL. Cylindrical hydrogel molds were constructed by cutting the Luer-Lock tips off of BD disposable 1 mL syringes. Gel precursor solution was added in 40 μL aliquots to the syringe molds and exposed to UV light at 4 mW/cm^2^ (365 nm) for 10 min. Gels were transferred to 24 well plates and rinsed three times with 500 μL PBS. One mL of OPC media per gel was added and the gels were cultured for up to 7 days, changing media every other day.

### 2.7 Nanoindentation

An Optics11 Chiaro nanoindenter with attached brightfield microscope was used to determine local elastic moduli of 1.5% NorHA hydrogels containing encapsulated cells (0.5 × 10^7^ cells/mL) and fibers (1 wt %) at culture day 5. Probe stiffness was 0.021 N/m and glass tip radius was 24.5 um. Measurements were performed when the spherical tip of the probe was located directly above a feature of interest (cell or fiber) or >10 μm from a feature of interest. Three hydrogel samples were used to collect measurements from both fiber-free hydrogels and fiber containing hydrogels. In fiber-free gels, probe measurements were collected above OPC clusters (*n* = 3 measurement replicates), or distant from any features (*n* = 6 measurements). In fiber-containing gels, probe measurements were collected directly above electrospun fibers (*n*= 4 measurement replicates), directly above an encapsulated OPC spheroid (*n* = 3 measurement replicates), or distant from any features (*n* = 3 measurement replicates).

### 2.8 Microplate Biomolecular Assays of Viability and Growth

Hydrogel samples with encapsulated OPCs were collected at days 1, 4, and 7 of culture with six sample replicates for each of the fiber-containing and fiber-free conditions (12 gels total). Hydrogels were separated into microtubes (1 gel per tube) containing 300 μL 1× passive lysis buffer (Promega) and frozen at −80 °C. Prior to conducting the assays, samples were retrieved from storage in the −80 °C freezer and thawed on ice for 1 h. After thawing, gels were homogenized using a hand-held pestle homogenizer followed by sonication for 10 s.

ATP concentrations of each sample were measured to assess the metabolic activity of OPCs within the hydrogel microtissues. Homogenized samples were pipetted with three pipetting replicates for each of six sample replicates per condition. In a white 384-well plate, 20 μL of PBS was combined with 5 μL of sample in each sample well. Twenty-five μL of CellTiter-Glo 2.0 reagent (CellTiter-Glo 2.0 Cell Viability Assay, Promega) were then added to each of the standard and sample wells. The plate was incubated at room temperature for 10 min, and luminescence was measured using a BMG ClairoStar microplate reader.

The concentration of DNA was measured in a similar way to the ATP assay. Homogenized hydrogel samples were pipetted with three pipetting replicates for each of six sample replicates per condition. For each sample, 5 μL of sample were added to 15 μL 1× TE buffer. The Pico Green reagent (Quant-iT PicoGreen dsDNA Kit, Invitrogen) was prepared by adding 50 μL of Pico Green dye to 10 mL 1× TE Buffer. 20 μL of dye was added to each of the standard and sample wells. The plate was incubated at room temperature for 10 min. Samples were excited at 480 nm and fluorescence was measured at 520 nm using a BMG Clariostar microplate reader. ATP values for each sample were normalized to DNA values, since DNA concentration scales with the number of cells. Resulting ATP/DNA ratios were then normalized to the day 1 no-fiber control.

### 2.9 Morphology Staining and Quantification of Maturation-Associated Cell Spreading

To assess cell morphology, gels were fixed and stained with DAPI and phalloidin at culture days 1, 4, and 7 to label nuclei and the f-actin cytoskeleton, respectively. Hydrogel microtissues were fixed in 4% paraformaldehyde solution for 1 h at 37 °C. Three 5 min washes with 3% bovine serum albumin (BSA) in PBS were conducted after fixation and between each subsequent step. Fixed hydrogels were permeabilized at room temperature in 0.1% Triton X-100 (Fisher Scientific) for 30 min at 4 °C with gentle shaking. Gels were stained overnight at 4 °C with 2 U/mL AlexaFluor 647 phalloidin (ThermoFisher) in PBS with 1% BSA. To label cell nuclei, gels were stained with 300 nM DAPI (Sigma) for 1 h at 4 °C with shaking. Following staining, gels were rinsed 4–6 times in 1 h washes with 3% BSA in PBS. Stained gels were stored in PBS followed by imaging with a Leica SP8 confocal. Z stack images were collected at 20× magnification to a Z depth of 300–500 μm.

To quantify cell spreading, 9 z-stack images were taken for each of the fiber-free and fiber-containing conditions (3 images per gel across 3 gel replicates). Multiple maximum intensity projections of Z depth interval 20.56 μm were constructed to span the entire Z depth of the hydrogel (300–500 μm). Every second interval was quantified to avoid potential double-counting of cells spanning multiple intervals. For each Z interval, DAPI and phalloidin channels were separated and converted to 16-bit images. The brightness of each channel was adjusted using the Auto Brightness function in FIJI ImageJ.^46^ Images were processed using the Auto Threshold function. The Despeckle function was then applied to remove background noise. The Analyze Particles function was used to quantify signal area for both the DAPI and phalloidin channels. Signal area from particles less than 20 pixels^2^ in size was excluded to avoid counting noncellular signal. Phalloidin signal area was then normalized to DAPI signal area to determine the average phalloidin signal area per cell.

### 2.10 Cell Proliferation Staining, Imaging, and Quantification

MADM OPC proliferation in 1.5 wt % NorHA gels was measured on days 1, 4, and 7 by staining hydrogels using the Click-iT EdU Imaging kit (Life Technologies). Briefly, cell-laden hydrogels were incubated in EdU solution for 1 h at 37 °C. Microtissues were then fixed in 4% paraformaldehyde solution for 1 h at 37 °C. Two 5 min washes with 3% bovine serum albumin (BSA) in PBS were conducted after fixation and between subsequent steps. Fixed hydrogels were permeabilized at room temperature in 0.1% Triton X-100 for 30 min, then incubated in Click-iT reaction buffer for 30 min. To label cell nuclei, gels were stained with 300 nM DAPI (Sigma) for 1 h at 4 °C with shaking. Following staining, gels were stored in PBS followed by imaging with a Leica SP8 confocal.

Z stack images were collected at 20× magnification to a Z depth of 300–500 μm. 9 z-stack images were taken for each of the fiber-free and fiber-containing conditions (3 images per gel across 3 gel replicates). A maximum intensity projection was made over the entire Z depth (300–500 μm). DAPI and EdU channels were separated and converted to 8-bit images. For each channel, brightness was adjusted using the Auto Brightness function in ImageJ. Images were processed using the Auto Threshold function in ImageJ. The ImageJ Watershed function was applied to better distinguish the signal of individual cells within clusters. The Despeckle function was then applied to remove background noise. The Analyze Particles function was used to quantify signal area for both the DAPI and EdU channels. Signal area from particles less than 8 pixels^2^ in size was excluded to avoid counting signal area resulting from cell debris. EdU signal area was then normalized to DAPI signal area to determine the average proliferation signal area per cell.

### 2.11 Nascent Extracellular Matrix Deposition

Nonspecific ECM protein labeling was conducted according to previously established protocols.^26^ Briefly, cell-containing gels were cultured in a modified media consisting of glutamine-, methionine- and cystine-free high glucose DMEM (Gibco) supplemented with 0.201 mM cystine, 0.151 mM methionine, 100 μg/mL sodium pyruvate, 4 mM glutamine, N2 supplement, B27 supplement, 1% penicillin-streptomycin, and 50 μM azidohomoalanine (AHA, Click Chemistry Tools). Newly synthesized proteins incorporated AHA as an analogue to methionine. Medium was replenished every second day. Gels were cultured for a period of 5 days. To stain for nascent protein deposition, hydrogels were washed twice with 1% BSA in PBS, followed by 30 min staining with 15 μM DBCO-647 (Click Chemistry Tools) at 37 °C/5% CO_2_. DBCO bound specifically to AHA in newly synthesized proteins, allowing for visualization of nascent protein secreted externally to the cells. After three washes with 1% BSA-PBS, hydrogels were fixed in 4% paraformaldehyde for 1 h at room temperature (RT) followed by three washes in 1% BSA-PBS. Cell membrane was stained for 40 min in CellMask Orange (1:1000, ThermoFisher) followed by three washes in 1% BSA-PBS. Samples were stored in 1% BSA-PBS at 4 °C.

### 2.12 Laminin and Fibronectin Immunostaining

Cell-containing gels were cultured in normal growth media, which was replenished every second day. To stain for specific ECM proteins, gels were fixed in paraformaldehyde for 1 h at 37 °C/5% CO_2_, followed by 3 washes in 2% BSA-PBS. A blocking step was conducted in 2% BSA-PBS at RT, and gels were incubated with primary antibodies (rabbit antilaminin α5, 1:100, ThermoFisher or rabbit antifibronectin, 1:250, Abcam) in 2% BSA-PBS for 12 h at 4 °C. Gels were washed twice, followed by a 6–8 h rinse in 2% BSA-PBS. Secondary antibody (donkey antirabbit 647, 1:200, Abcam) was applied in 2% BSA-PBS at 4 °C for 12 h. Plasma membrane was stained with CellMask Orange (1:1000, ThermoFisher) for 35 min, followed by 3 rinses with 2% BSA-PBS. Gels were stored in 2% BSA-PBS at 4 °C.

### 2.13 ECM Deposition Image Quantification of Nascent Total Protein and Laminin/Fibronectin

4–6 z-stack images were taken for each of the fiber-free and fiber-containing conditions (2 images per gel across 2–3 gel replicates). Z stacks were taken at 20× magnification in intervals of 2 μm to a depth of 250–400 μm. For each Z interval, CellMask and DBCO or protein antibody channels were separated and processed using the Otsu thresholding function in FIJI ImageJ.^46^ The Despeckle function was then applied to remove background noise. A dedicated ImageJ macro was used for automated quantification of signal area of both the CellMask and protein channels for each Z interval in the sample.^47^ Protein signal area was then divided by CellMask signal area to determine the amount of extracellular matrix deposition normalized to cell surface area.

### 2.14 Statistical Analysis

Error bars for rheological characterization represent standard deviation with error propagation. ATP and DNA data were analyzed using two-sample *t* test with a α-value of 0.05. Morphology and proliferation signal areas were analyzed using 2-way ANOVA with a α-value of 0.05 to assess signal area with respect to fiber presence and time point. Two-sample *t* tests (α-value of 0.05) were subsequently conducted to compare signal areas for fiber-containing and fiber-free gels at each time point. ECM deposition results were analyzed using 3-way ANOVA with a α-value of 0.05 to assess amount of ECM deposition with regards to 3 factors (protein type, fiber presence and time point). Two-sample *t* tests (α-value of 0.05) were subsequently used to conduct pairwise comparisons between two factors (fiber presence and time point). Box plots cover the second and third data quartiles with error bars covering the first and fourth quartiles. Box plots also include marks for mean (x) and median (bar) data values.

## 3 Results and Discussion

### 3.1 Fiber Characterization

**Figure 1:**
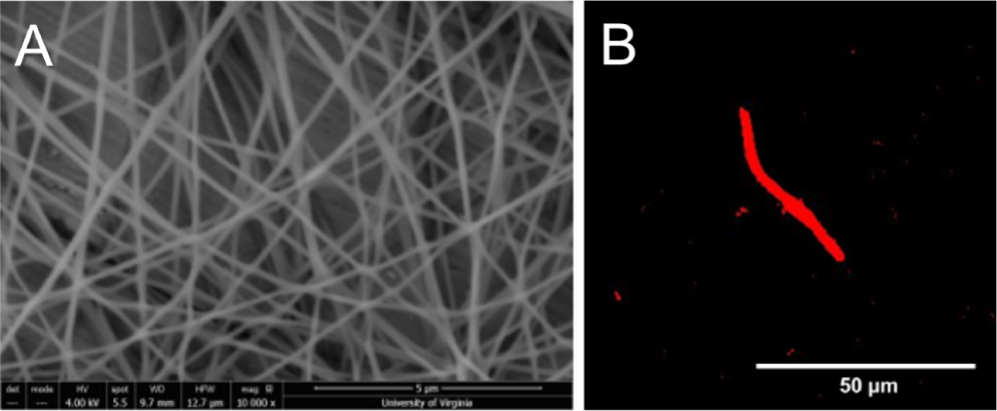
Characterization of dry and swollen electrospun fibers. (A) SEM images of dry electrospun fiber mats (scale bar = 5 μm). Dry fiber diameter was quantified at 181 ± 4 nm. (B) Representative fluorescent images of swollen hydrated fibers (isolated fibers in 2D). Swollen fiber diameter was quantified as 2680 ± 170 nm.

The diameter of dry, electrospun MehA fibers was determine using scanning electron microscopy (SEM) (Figure 1A). The fiber diameters were uniform both along the length of individual fibers and across different fibers, with an average dry fiber diameter of 181 ± 4 nm, similar to MeHA fibers produced by other groups using similar electrospinning conditions.^37^

Fibers were swollen to equilibrium in PBS and mechanically separated by repeatedly passing fiber solution through 18G and 21G needles to break up fiber mats. Swollen fiber stock solution was examined in 2D via fluorescent microscopy to measure the swollen fiber diameter (Figure 1B). The swollen fiber diameter was found to be 2670 ± 170 nm. Within the central nervous system, most myelinated axons typically range from 0.3 to 2 μm in diameter.^29^ Previous 2D in vitro studies with oligodendrocyte precursor cells (OPCs) have shown that fibers with diameters greater than 0.4 μm are preferentially myelinated, with OPCs favoring fibers in the 2–4 μm range.^29^ Therefore, our fiber size corresponds to the diameters of mature CNS axons found in native neural tissue.

### 3.2 Biomaterial Stiffness Characterization

**Figure 2:**
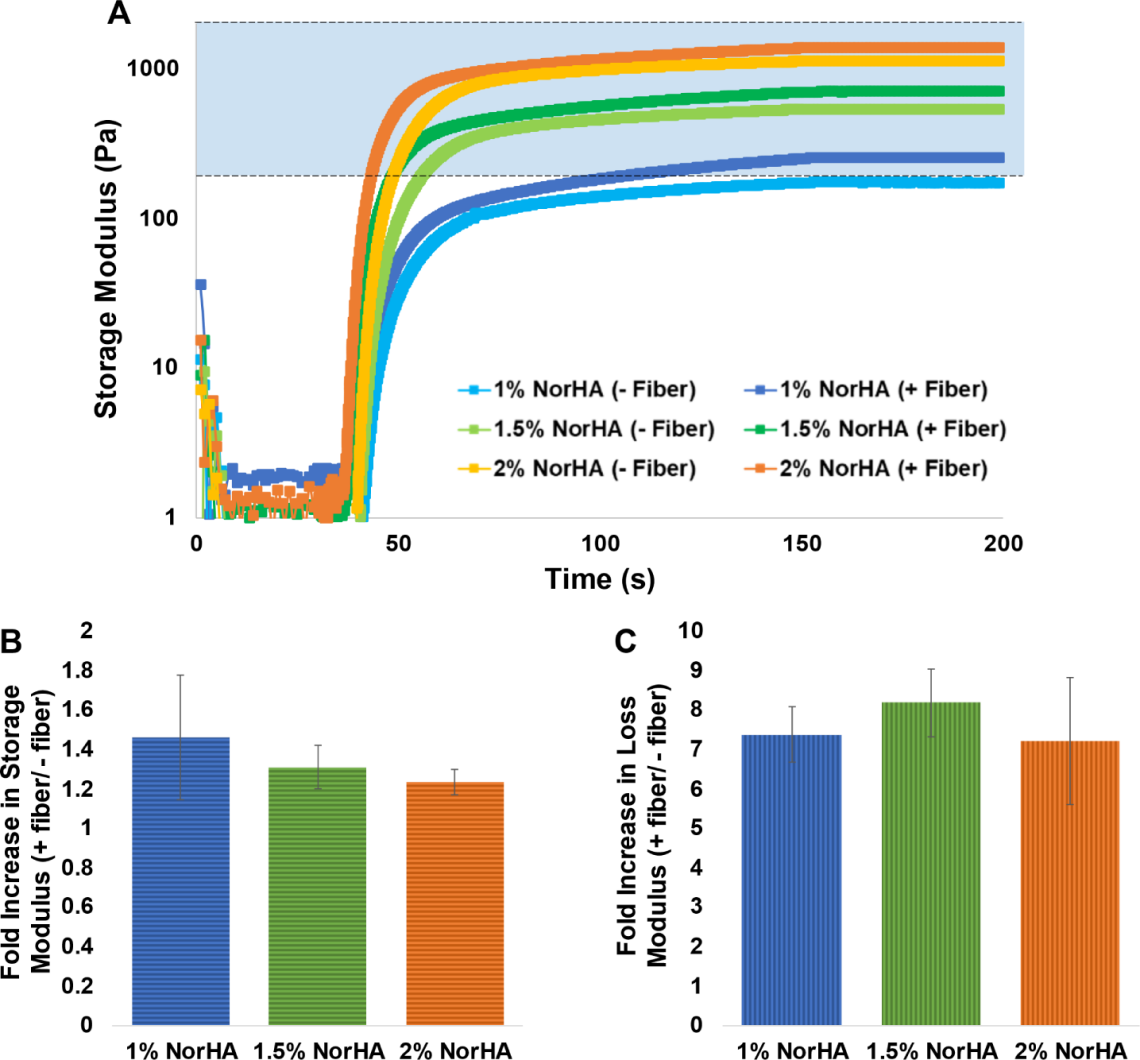
Rheological characterization of NorHA gels ± fibers. (A) Time-sweep oscillatory shear rheology measurements for on-stage gelation of 1–2 wt % NorHA gels. Each trace represents the average of multiple trials (*n* = 4). The shaded blue region represents the storage modulus range of native brain tissue (*G*′ = 200–2000 Pa). Plateau storage modulus increases with increasing NorHA concentration. Fiber containing gels at each condition had higher storage moduli than no-fiber controls with equivalent NorHA concentrations. (B) Fold increase in final storage moduli of fiber-containing gels (*n* = 4) as compared to control gels (*n* = 4) of equivalent NorHA concentration. The addition of fibers resulted in a 20–40% increase in final storage modulus, with the percent increase being more pronounced in softer gels. Fold increase in storage modulus was not significantly different between the three tested NorHA concentrations. (C) Fold increase in final loss modulus of fiber-containing gels (*n* = 4) as compared to control gels (*n* = 4) of equivalent NorHA concentration. Fiber containing gels have a 7–8 fold increase in loss modulus as compared to no fiber controls. Fold increase in loss modulus was not significantly different for any of the three tested NorHA concentrations.

Hydrogels containing between 1 and 2 wt % NorHA (functionalization 24–30%) accurately reproduced the range of storage moduli present in native brain tissue (200–2000 Pa) (Figure 2A).^22,48,49^ Although two different methods of NorHA synthesis were used, both methods produced comparable products with equivalent levels of functionalization (Figures S1 and S2). Inclusion of cross-linked electrospun fibers at a concentration of 1 wt % in the gel precursor solution resulted in a 20–40% increase in plateau storage modulus as compared to fiber-free hydrogels, with the effects of fiber addition being most pronounced in the most compliant (1% NorHA) gel condition (Figure 2B). Because the fibers are solids with a higher storage modulus than the surrounding hydrogel, inclusion of fibers therefore results in a small (though statistically significant) increase in the overall bulk modulus. However, in all conditions, the difference in moduli resulting from fiber inclusion was much smaller than the moduli difference due to different polymer weight percent conditions. For example, the most pronounced difference due to fiber inclusion (between 1% NorHA + fiber and 1% NorHA – fiber) was 80 Pa, while the difference between 1% and 1.5% NorHA was an order of magnitude larger, at 365 Pa. We have previously demonstrated the change in storage modulus between HA hydrogels of different wt % to impact OPC proliferation, but observed no effect on cell morphology or process extension.^22^ Inclusion of fibers was also associated with a several-fold increase in plateau loss modulus as compared to gels without fibers (Figure 2C). The increase in loss modulus (Figure 2C) is explainable as fibers dispersed throughout the gel increase its viscous nature, which can be observed prior to cross-linking.

**Figure 3:**
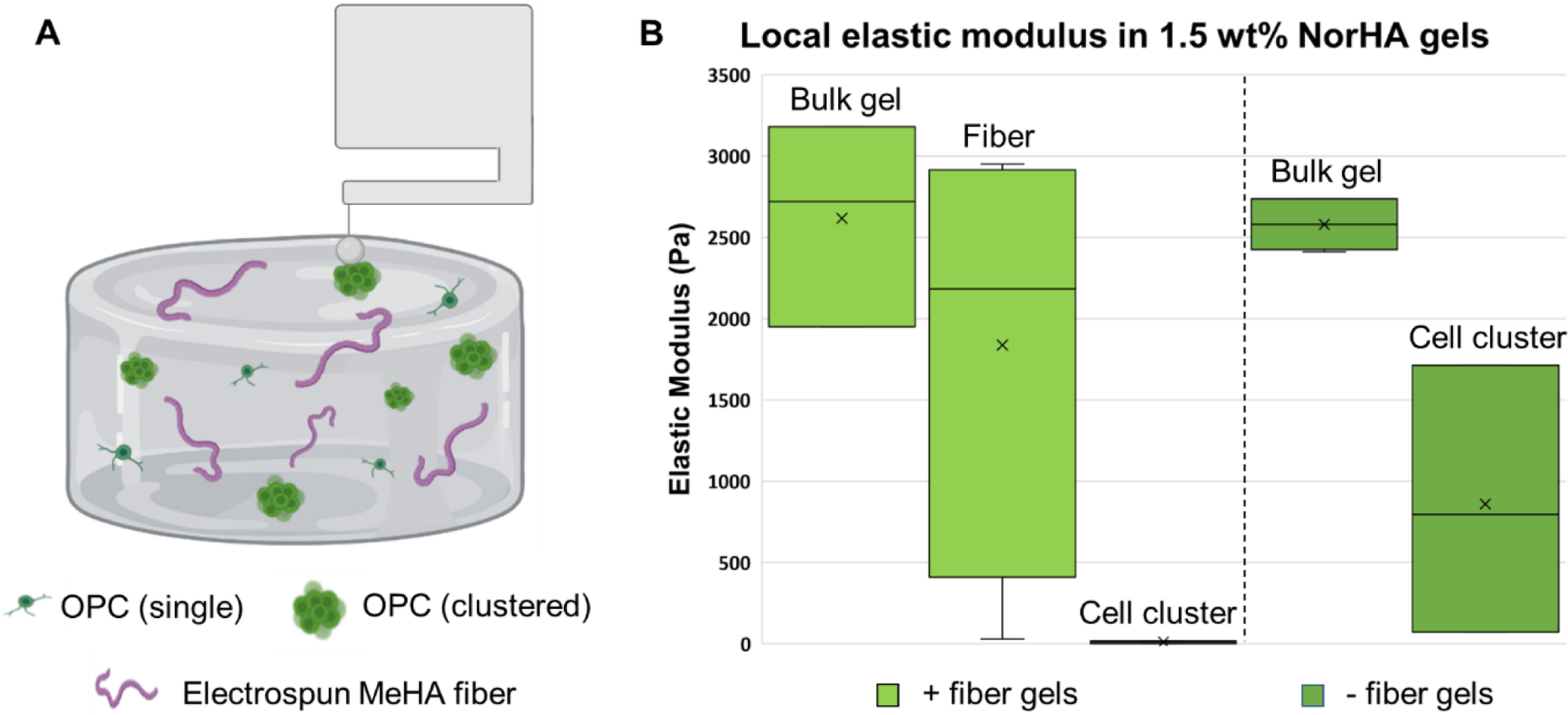
Mechanical characterization of local elastic modulus properties using nanoindentation. (A) A nanoindentation probe was used to determine local elastic moduli of 1.5% NorHA hydrogels containing encapsulated cells (0.5 × 10^7^cells/mL) and fibers (1 wt %). (B) Local elastic modulus readings for 1.5% NorHA hydrogels (*n* = 3 gel replicates, 3–4 measurements per gel). The midline of the boxes represents the median, while the x in the box represents the mean. The boxes represent the range between the first and fourth quartiles, and the whiskers indicate the minimum and maximum values. Elastic modulus measurements taken above cell clusters were found to be significantly lower compared to those for the bulk gel. Measurements taken above fibers did not significantly differ from measurements of the bulk gel.

To determine the effects of fibers on local elastic modulus, single point nanoindentation was also performed on 1.5 wt % NorHA hydrogels containing both cells and fibers. The nanoindentation probe was positioned directly above various features of the hydrogel system (cells, fibers, or bulk gel) to determine local elastic modulus near these features (Figure 3A). Nanoindentation measurements atop cell clusters had a wide range of elastic moduli, due to variability of cell cluster depth. However, in both hydrogel types (fiber-containing or fiber-free) the presence of underlying cell clusters significantly reduced the local elastic modulus measurement. We attribute this to OPCs’ low elastic modulus and their local disruption of the hydrogel mesh (Figure 3B).^50^ Similarly, measurements taken atop fibers had a large variance in elastic modulus, due to variation of fiber depth (Figure 3B). However, while bulk gel readings in the fiber-containing gels showed a wider range compared to equivalent measurements in no-fiber gels, the mean elastic modulus was equivalent in both conditions (Figure 3B). This suggests that, although fibers consistently increase bulk storage modulus of the gel (Figure 2A), local elastic modulus of the gel is largely unaffected. These materials characterization results suggest that while fiber presence has an impact on material properties, this impact is local and limited to a range within 10 μm from the fibers. Overall, material properties remain comparable between fiber-free and fiber-containing conditions.

### 3.3 Assessment of Cell Spreading and Morphology Changes Associated with Maturation

**Figure 4:**
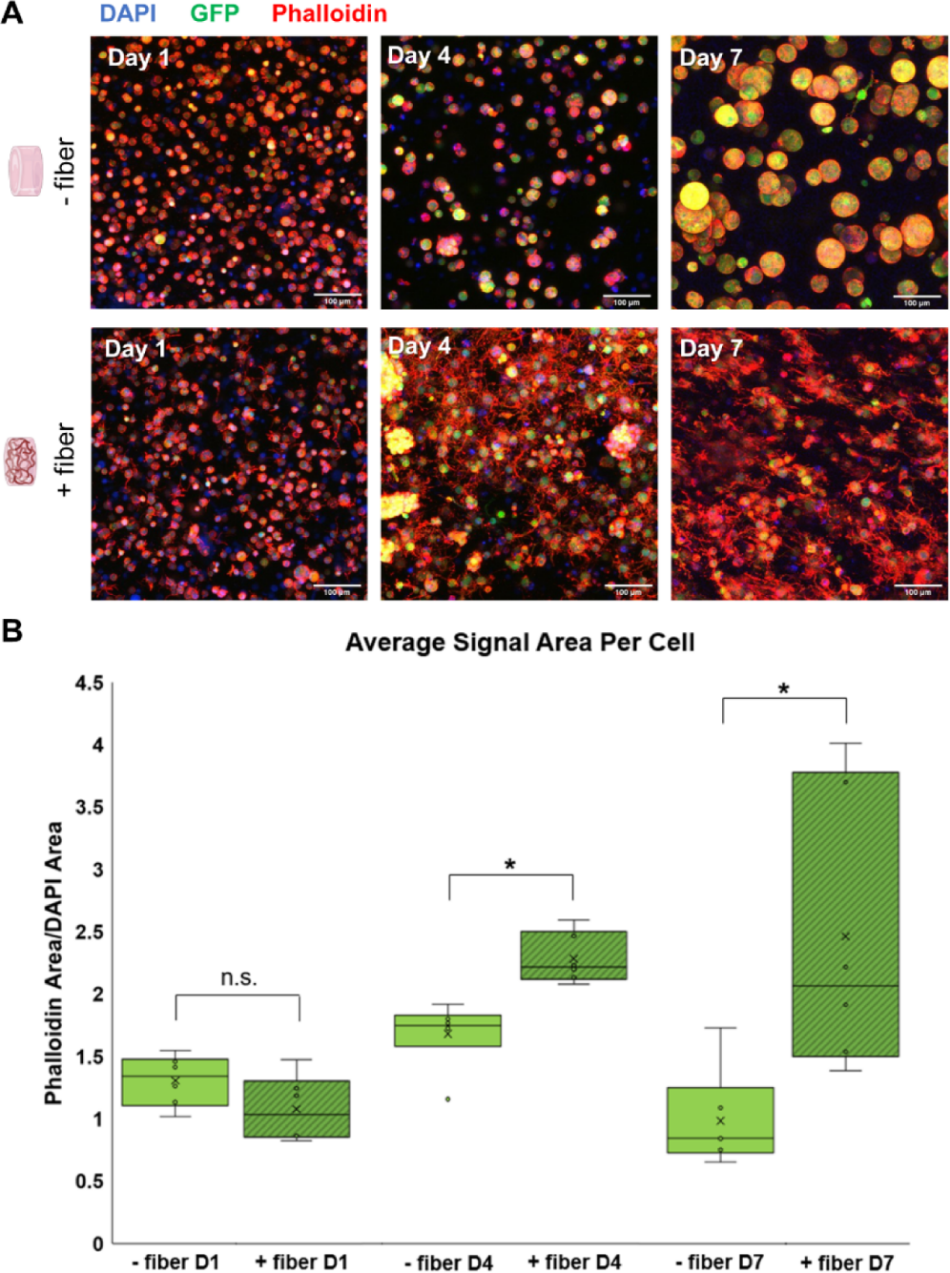
OPC morphology in fiber-free controls and fiber-containing gels. (A) Maximum intensity projection of OPCs encapsulated in fiber-free (top row) control gels and fiber-containing (bottom row) gels after 1, 4, and 7 days of culture (*n* = 3 gel replicates, with 3 imaging replicates per gel). Cells are stained with DAPI (blue, nuclei) and phalloidin (red, cytoskeleton), and express green fluorescent protein (green, cytoplasm). In the absence of fibers, cells retained a rounded and immature morphology, and grow mostly as clonal spheroids. Cells in fiber-containing gels extended numerous processes, an indicator of OPC maturation. (B) Average phalloidin signal area per cell was initially similar for both conditions at culture day 1 (D1) but increased significantly for cells in fiber containing gels at D4 and D7 time points. n.s. indicates no significant difference between conditions, while * indicates a significant difference (*p* = 0.05). Boxes cover the second and third data quartiles with error bars covering the first and fourth quartiles. Mean values are indicated by an x and median values with a bar, while individual data points are represented by circles.

To evaluate the effect of fiber presence on the morphology of encapsulated OPCs, gels from both the fiber-free and fiber-containing groups were fixed and stained at days 1, 4, and 7 of culture. Maximum intensity projections of phalloidin-labeled cells in the no-fiber control gels show that cells grow and form OPC spheroids through localized proliferation over a 7-day culture period but maintain a rounded and immature morphology (Figures 4A and S4). By contrast, cells in fiber-containing gels consistently demonstrate a more mature morphology, indicated by the cells’ extension of numerous processes (Figures 4B and S4).

Encapsulated OPCs initially exhibit similar cytoskeletal volumes, as determined by phalloidin/DAPI ratios, across both conditions at day 1 of culture (with mean signal area ratios of 1.08 and 1.30 for fiber-containing and fiber-free gels, respectively). However, cells in fiber-containing gels displayed significantly larger cytoskeletal area ratios by day 4 of culture. The difference between conditions continued to increase with time and was most pronounced at day 7 of culture, where fiber-containing gels had a mean phalloidin/DAPI signal area ratio of 2.47, compared to 0.99 for cells in gels without fibers (Figure 4C). ANOVA indicated that both fiber presence and time point significantly affected cell size, with fiber-containing gels and gels at later time points exhibiting larger signal areas. Because the size of the main cell body remained similar across conditions, we attribute the increase in signal area to increased levels of process extension in fiber-containing gels over time.

This rounded OPC spheroid morphology in fiber-free gels is consistent with previous work in both NorHA and polyethylene glycol-based hydrogel systems.^22,23^ Their rounded appearance is consistent with immature OPCs (Figure 4A). By contrast, process extensions, a hallmark of cell maturation, appeared throughout the entirety of fiber-containing gels. Notably, this result occurred in standard proliferation media; no specific soluble cues were added or subtracted to promote differentiation. Changes in morphology across conditions cannot be attributed to differences in bulk modulus, since previous experiments by our group have shown that OPCs grown in fiber-free gels across a wide range of storage moduli produce no observable process extensions.^22^ These results indicate that topographical cues alone are capable of prompting OPC maturation in 3D despite strong mitogenic input.

Previous work by other groups has demonstrated that OPCs and oligodendrocytes in 2D culture are able to extend processes and wrap around axon-mimicking fibers to deposit myelin.^29,30^ These studies found that fiber cues with diameters similar to mature axons (2.0–4.0 μm) were preferentially ensheathed by about 60% of OPCs and oligodendrocytes.^29^ Our results are consistent with these studies, as only those OPCs grown in the hydrogels containing micron-diameter fibers extended substantial processes, and almost all cells extended processes (Figure 4A,B). However, our culture system provides the novel advantage of encapsulating both OPCs and fiber cues fully in 3D in an ECM-mimicking hydrogel (as opposed to culturing OPCs atop fibers alone). Furthermore, our fibers are hydrogels which more accurately recapitulate axon mechanics than stiff polystyrene. Our results may therefore more accurately model OPC behavior by better mimicking conditions in native tissue, including 3D cell-matrix interactions, bulk storage modulus, and topographical features. This system may provide certain advantages for substantial scaleup beyond 2D surfaces.

### 3.4 Effects of Fibers on Cell Proliferation and Metabolic Activity

**Figure 5:**
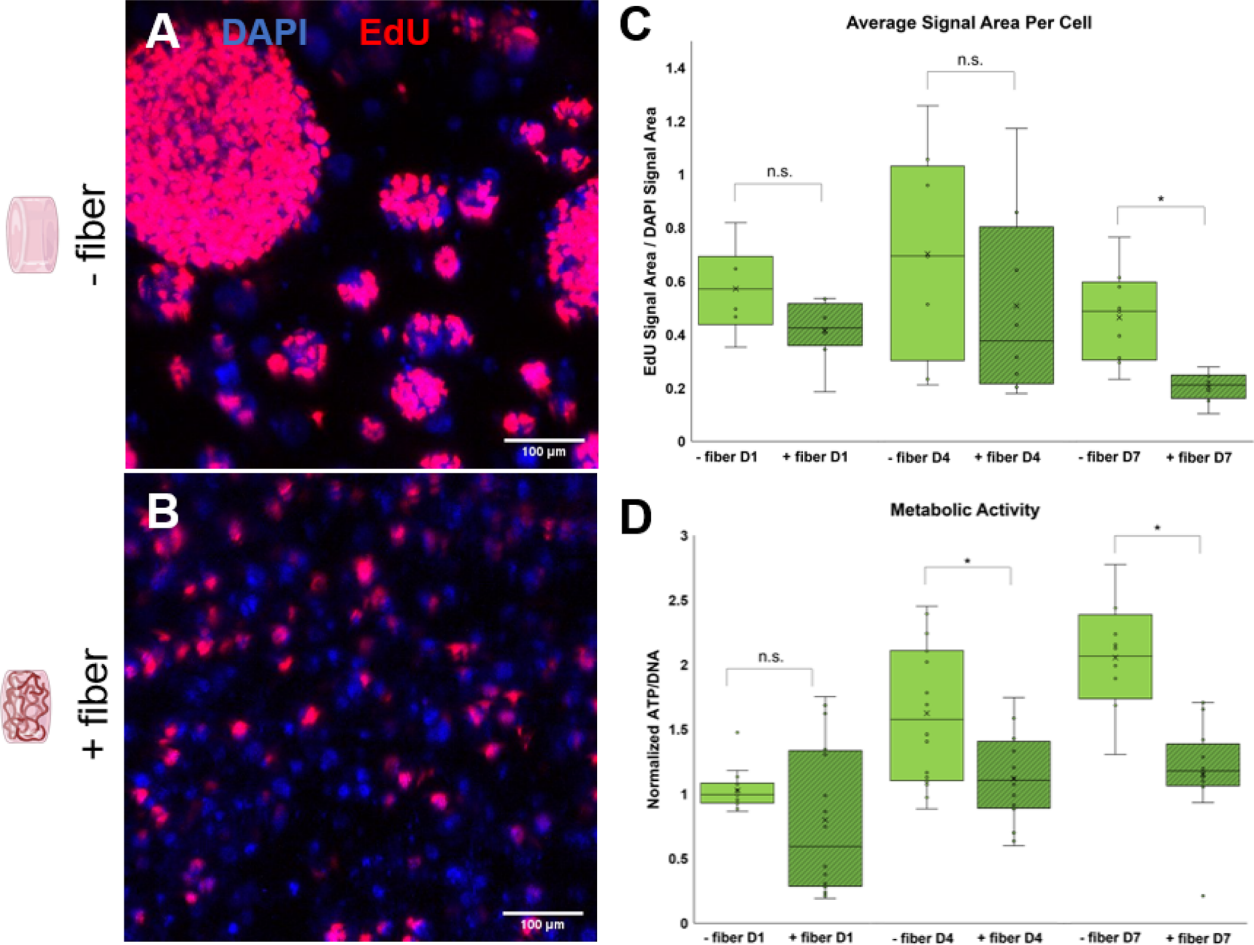
OPC proliferation in fiber-free controls as compared to fiber-containing gels. (A) OPCs encapsulated in control gels without fibers resulted in a high proportion of proliferating cells at D7, and many large spheroids. (B) Cells in fiber-containing gels do not form large clusters and proliferate to a lesser extent as compared to cells in no-fiber controls. (C) Fraction of actively proliferating OPCs based on normalized EdU/DAPI signal area (*n* = 3 gel replicates per condition, with 3 imaging replicates per gel). While proliferation was initially similar at D1 and D4, fiber-containing gels trended toward lower proliferation rates throughout the study and exhibited significantly lower proliferation levels at D7. n.s. indicates no significant difference between conditions, while * indicates a significant difference (*p* = 0.05). (D) Average metabolic activity, determined by ATP concentration normalized to DNA concentration (*n* = 6 gel replicates per condition). Metabolic activity was initially similar across both conditions at D1, but was significantly higher for cells in no-fiber controls at D4 and D7 time points, consistent with a higher proportion of proliferating progenitor cells (*p* = 0.05).

Similarly, proliferation in both the control and experimental groups was evaluated at days 1, 4, and 7 of culture with DAPI/EdU staining (Figures5A,B and S5). OPC proliferation appears similar across both the fiber-free control and fiber-containing conditions at the D1 time point (with mean EdU/DAPI ratios of 0.57 and 0.42, respectively), and peak at a similar level on D4 (mean EdU/DAPI ratios of 0.7 and 0.5). However, proliferation for both groups declines after D4, and the proliferation ratio for fiber-containing gels was found to be statistically lower at the D7 time point (mean EdU/DAPI = 0.21) as compared to no-fiber controls (mean EdU/DAPI = 0.46) (Figures 5C and S6). Overall, ANOVA indicated that both fiber presence and time point significantly affected cell proliferation. Metabolic activity, as measured by normalized ATP/DNA concentration, was initially similar across conditions at D1, but was significantly lower in fiber-containing gels at D4 and D7 time points (Figure 5D). The notable decrease in proliferation in fiber-containing gels (Figure 5C) is consistent with our observations of cell morphology over a seven-day culture period; cells in the absence of electrospun fiber cues retained an immature, proliferative state, while OPCs in the presence of fibers proliferated less and instead matured and differentiated toward postmitotic phenotypes with a lower metabolic turnover. The observed changes in metabolic activity are also consistent with literature, as OPCs are known to have heightened metabolic demands while actively proliferating.^51−53^ It therefore follows that cells in fiber-free gels demonstrate higher levels of metabolic activity in conjunction with their higher rates of proliferation.

### 3.5 Impact of Fiber Presence on Extracellular Matrix Deposition and Distribution

**Figure 6:**
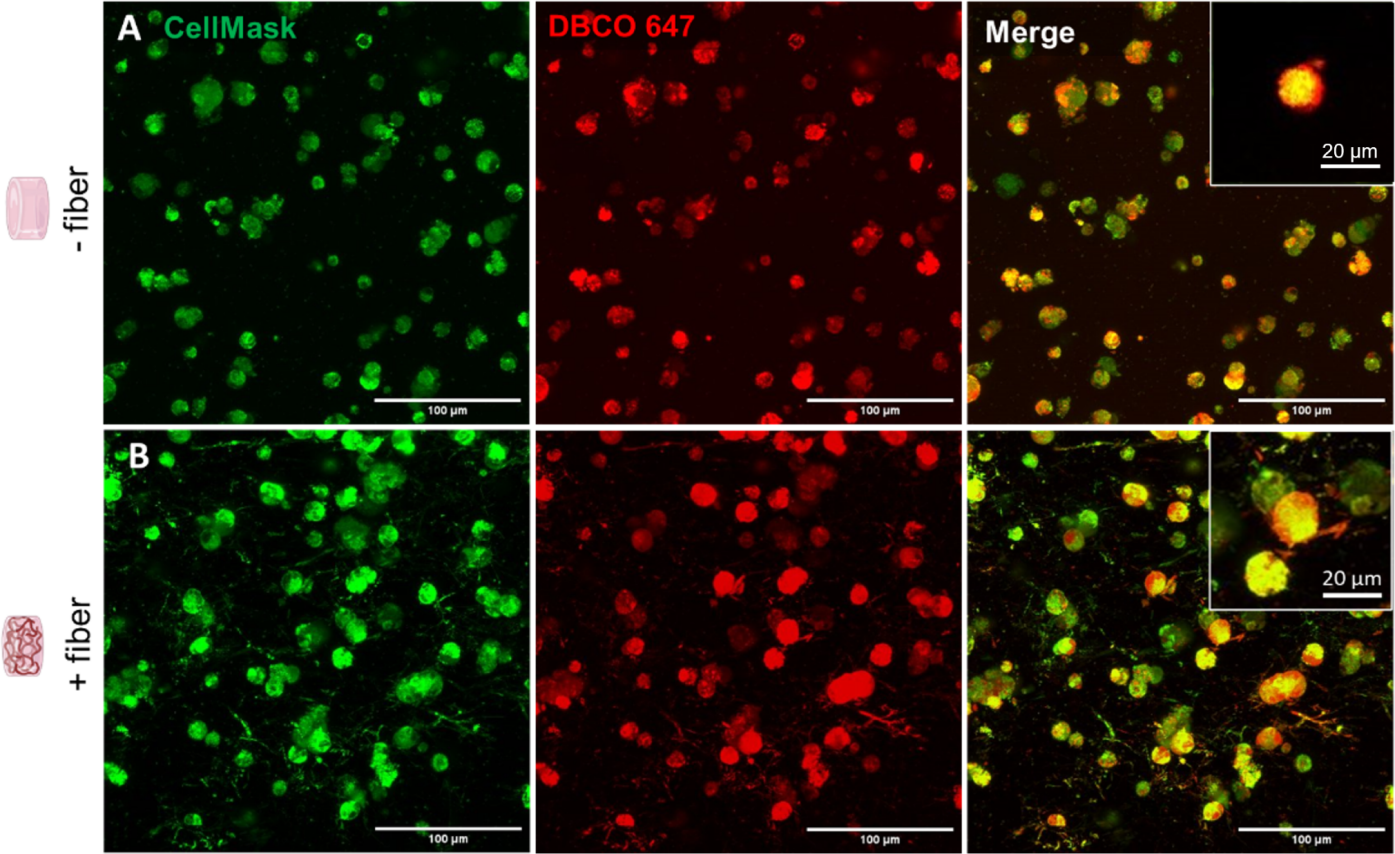
Maximum intensity projections of nascent extracellular matrix deposition around OPCs in fiber-free (A) and fiber-containing (B) samples at culture day 5. Merge projections reveal that in the fiber-free condition, extracellular matrix proteins were deposited around the outside of cells and cell clusters in a thin spherical shell (A, inset of a single Z slice). In the fiber-containing condition, ECM deposition surrounded the main cell bodies, but was also seen to extend along the lengths of cell process extensions (B, inset).

Since our hydrogels do not include specific integrin-binding sequences, we hypothesized that, like other cells, OPCs may be depositing extracellular matrix proteins to remodel their local environment. We selected a single day 5 time point for examination of nascent ECM deposition as previous work has demonstrated this is a sufficient amount of time in HA hydrogels to capture deposition and distribution.^26^ Nascent ECM deposition occurred in both the fiber-containing and fiber-free conditions. In the fiber-free control gels, cells retained a rounded morphology and ECM deposition occurred in a thin spherical shell surrounding the outside of cells and cell clusters (Figure 6A). In the fiber-containing condition, ECM deposition occurred in a similar pattern surrounding the main cell bodies, but also occurred in association with cell process extensions (Figure 6B).

**Figure 7:**
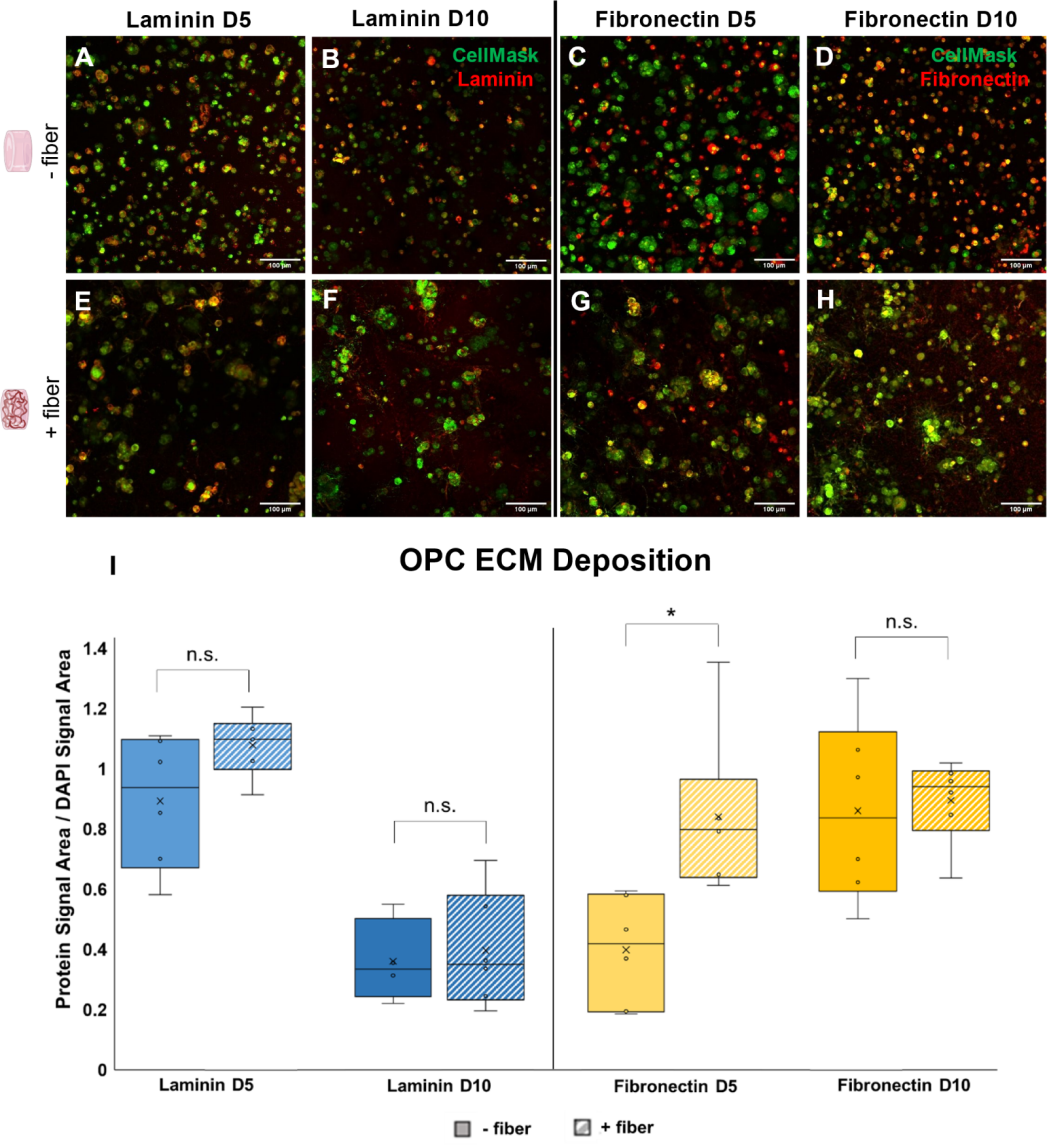
Maximum intensity merge projections of laminin (LN) and fibronectin (FN) matrix deposition around OPCs in fiber-free (A–D) and fiber-containing (E–H) samples at culture days 5 and 10. CellMask (green) was used to label cell membranes, while immunostaining (red) was used to assess for the presence of laminin or fibronectin. Staining for specific ECM proteins reveals similar patterns of deposition as were observed in the nonspecific ECM deposition assay. (I) Quantification of protein signal area for laminin and fibronectin (*n* = 2–3 gel replicates, with 2 imaging replicates per gel). Laminin deposition decreases between time points for both fiber-containing and fiber-free conditions, while fibronectin deposition increases between time points for fiber-free gels and remains consistent between time points for fiber-containing gels.

In order to assess the composition of ECM deposits, immunostaining was performed for two common ECM proteins of the central nervous system, laminin and fibronectin. These proteins were chosen due to their previous implication as part of the OPC secretome.^27,28^ The time points were chosen at an intermediate time that directly corresponds to the nascent ECM deposition data (day 5). A second time point was selected to determine how specific ECM proteins may vary as a function of time, after cultures have had the opportunity to undergo more extensive structural maturation (day 10). Based on signal area in immunostained max projections, OPCs deposited laminin and fibronectin in fiber-free and fiber-containing gels in similar amounts (Figure 7).

While both laminin and fibronectin have been implicated in the OPC secretome, the overall composition and distribution of nascent OPC ECM deposition is understudied.^27,28^ Additionally, the timeline of protein deposition, and how it varies over the course of cell differentiation, remains poorly understood. We confirmed that both laminin and fibronectin deposition occurred in hydrogels across all tested conditions (Figure 7). These results suggest that not only do OPCs synthesize and locally deposit their own matrix in these hydrogels, but also that the presence of fibers directs cell morphology, which in turn influences the pattern of extracellular matrix deposition.

In general, the relative amount of deposition is unaffected by the presence of fibers; although more fibronectin deposition was observed in the fiber-containing condition at day 5, there was no significant difference in fibronectin deposition at culture day 10 or in laminin deposition at either time point. ANOVA did indicate that there was a significant difference between time points, with laminin area decreasing and fibronectin area increasing between days 5 and 10. This indicates that the majority of laminin deposition occurs before day 5 of culture, with minimal deposition occurring between days 5 and 10. By contrast, fibronectin deposition continues to occur past day 5 in fiber-free gels. Overall, both proteins were deposited in similar amounts but the timeline of deposition was different. Laminin was deposited earlier and decreased over time, whereas fibronectin levels remained steady or increased.

While our data indicates that ECM proteins laminin and fibronectin are being deposited, the impacts of this deposition on cell behavior remain unclear. However, we can speculate based on other work.^26,54^ A similar study by Loebel et al. using human mesenchymal stromal cells (hMSC) has shown nascent protein deposition occurring in a thin spherical shell, aligning with results observed in our fiber free-condition.^26,54^ Loebel et al. further explored cell-ECM interactions using both soluble RGD inhibitors and function-perturbing antibodies, and discovered that both cell spreading and mechanosensing decreased when cell-ECM interactions were blocked, suggesting cell-ECM interaction played a role in these processes.^26^ Similar experiments could be conducted in future work to determine if cell-ECM interactions play a role in OPC spreading and mechanosensing.

### 3.6 Model System Limitations

Our model system was constructed to be representative of native tissue, both in terms of storage modulus and fiber diameter, and provide information about how these variables may impact OPCs specifically. However, some limitations do exist. The OPCs used in these experiments are from a mouse glioma-derived cell line which expresses many OPC markers but contains gene knockouts which prevent complete differentiation into myelinating OLs.^55^ As such, these cells are not fully representative of the differentiation capability of primary or endogenous OPCs. Additionally, fibers were incorporated at a low concentration (1 wt % based on dry mass), which is well below typical axonal density in the CNS. This low fiber density is logistically efficient since it enables synthesis of a large number of hydrogel samples without an overwhelming amount of labor-intensive fiber spinning. The low fiber density also retains the optically transparent nature of NorHA hydrogels since the refractive index is only minimally different than a fiber-free hydrogel. Despite these limitations, the system represents a promising first step toward modeling OPC response to axonal topography in the CNS, and it is encouraging to note the dramatic morphological response which occurs in 3D even at low fiber concentrations. Furthermore, due to the tunability of our model design, future modifications can easily include other cell sources capable of complete differentiation (e.g., primary OPCs, NSCs, induced pluripotent stem cell-derived OPCs) as well as a higher encapsulation density of electrospun fibers.

## 4 Conclusions

Using NorHA hydrogels between 1 and 2 wt %, we were able to successfully replicate the storage modulus range of native CNS tissue (*G*′ ∼ 200–2000 Pa). Inclusion of electrospun fibers was found to affect the bulk storage modulus of the overall hydrogel, resulting in a small, but significant, increase in storage moduli across all conditions. However, differences resulting from fiber presence were much smaller than differences resulting from different polymer concentrations. Additionally, nanoindentation data indicate that differences in elastic modulus due to fibers were a localized effect and did not affect the elastic modulus far (>10 μm) away from fibers. Overall, bulk material properties remained comparable between fiber-free and fiber-containing gels.

The small differences in modulus between our hydrogel conditions has not previously demonstrated any impact on OPC morphology or differentiation.^22^ However, despite their similar bulk mechanical properties, fiber-free and fiber-containing gels resulted in strikingly different OPC morphologies. In fiber-free NorHA gels, OPCs primarily expand as multicellular spheroids, without notable morphological features or substantial process extensions. By contrast, the presence of electrospun fibers in the hydrogel dramatically changed OPC morphology, leading to extensive process elongation (an indicator of maturation) from almost all OPCs throughout the microtissue. Notably, this effect occurred even though cells were cultured under standard proliferation media, with no additional soluble factors included (or excluded) to promote differentiation. Due to their abundant process extensions, cells in fiber-containing conditions displayed correspondingly larger normalized phalloidin/DAPI signal areas as compared to those in fiber-free control gels. OPCs in fiber-free controls demonstrated higher levels of proliferation (as measured by normalized EdU/DAPI signal area) and metabolic activity (as measured by normalized ATP/DNA concentration). Thus, the inclusion of topographical cues in the form of electrospun fibers promotes OPC maturation, directing cell fate away from the OPC proliferation pathway they otherwise experience under these conditions.

While OPCs were known to secrete ECM proteins to remodel their local environment, information regarding the distribution, composition, and timeline of ECM deposition was limited. Using nonspecific ECM staining, we confirmed that OPCs deposit nascent ECM proteins in NorHA gels, and that ECM secretion occurred in a thin spherical shell surrounding cells or spheroids. ECM deposition also occurred along the lengths of process extensions in the fiber-containing condition. Immunostaining assays determined that both laminin and fibronectin were secreted by OPCs, and that deposition of both proteins occurred in similar amounts across conditions. However, deposition of laminin occurred primarily before culture day 5 and decreased on a per cell basis between culture days 5 and 10; by contrast, fibronectin deposition increased over this time period. These novel results demonstrate that nascent ECM deposition by OPCs varies significantly with time, while cell maturity has little effect on the amount or composition of secreted ECM.

## Supporting Information Available

^1^H NMR spectra for all compounds (Figures S1–S3), channel-separated views of day 7 morphology staining (Figure S4), proliferation staining at culture days 1, 4, and 7 (Figure S5), channel-separated views of day 7 proliferation staining (Figure S6), confocal images of OPC associating with electrospun fiber (Figure S7), overview of the fiber breakup process (Figure S8), and SEM micrographs of dry electrospun fibers (Figure S9) (PDF)
