# Supplementary material for: Guiding Oligodendrocyte Progenitor Cell Maturation Using Electrospun Fiber Cues in a 3D Hyaluronic Acid Hydrogel Culture System
Source: ACS Biomater Sci Eng. 2024 Dec 20;11(2):1025–37. doi: 10.1021/acsbiomaterials.4c01455 (PMC11815632; doi:10.1021/acsbiomaterials.4c01455)
Supplement: Supplementary file 1 — ab4c01455_si_001.pdf [file ab4c01455_si_001.pdf]

Guiding oligodendrocyte progenitor cell maturation using electrospun fiber cues in a 3D hyaluronic acid hydrogel culture system

Rachel A. Mazur[1], Kyle J. Lampe[1]

1 Department of Chemical Engineering, University of Virginia, Charlottesville, VA USA 22903

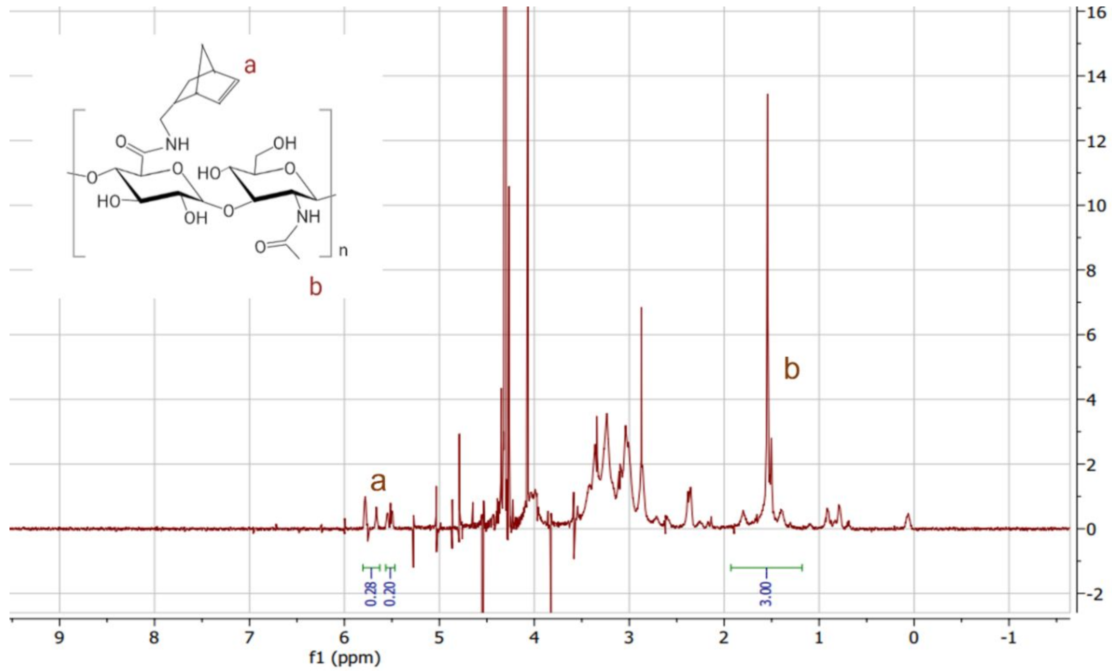

Figure S1. NMR spectra of 24% functionalized NorHA. Functionalization efficiency is calculated by normalizing the methyl peak area (b) to a value of 3. The average area of the two norbornene peaks (a) is then taken to determine the functionalization efficiency. Macromer product was generated using the BOP reaction scheme (Synthesis Method 1).

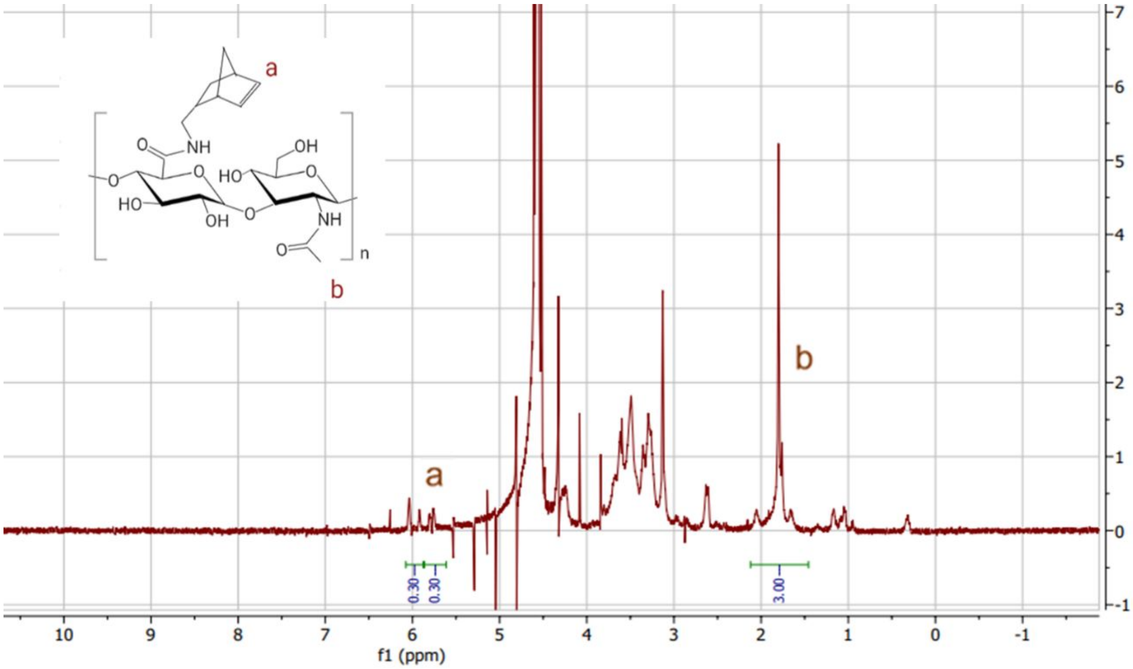

Figure S2. NMR spectra of 30% functionalized NorHA. Functionalization efficiency is calculated by normalizing the methyl peak area (b) to a value of 3. The average area of the two norbornene peaks (a) is then taken to determine the functionalization efficiency. NorHA macromer was synthesized using the DMTMM protocol (synthesis method 2). Despite the difference in synthesis methods, both BOP and DMTMM synthesis mechanisms produced equivalent products with comparable levels of norbornene functionalization.

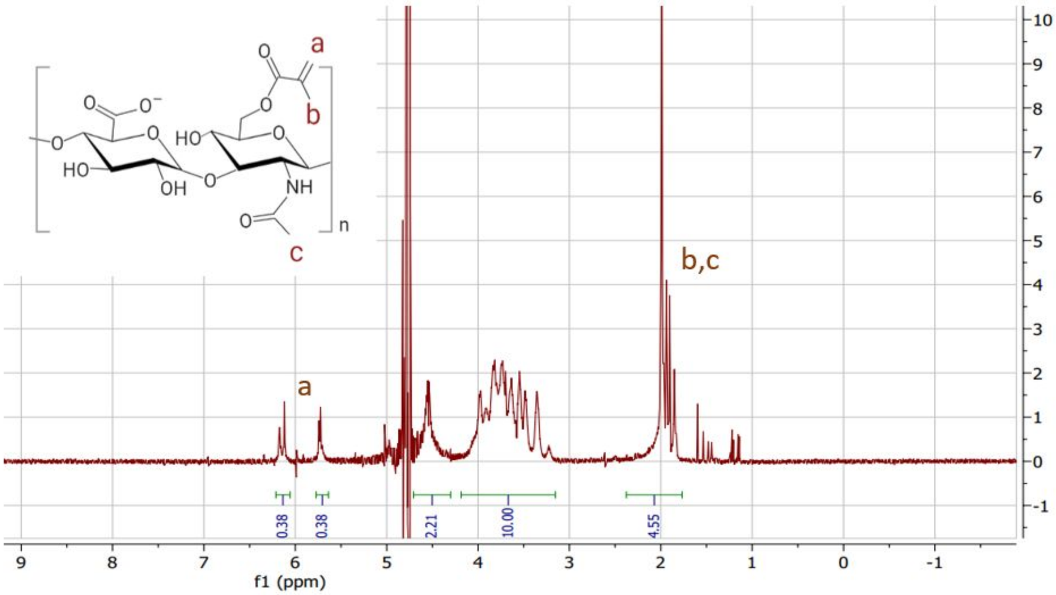

Figure S3. NMR spectra of 38% functionalized MeHA. Functionalization efficiency is calculated by normalizing the backbone peak area to a value of 10. The methacrylate peak areas (a) are then averaged to determine the functionalization efficiency.

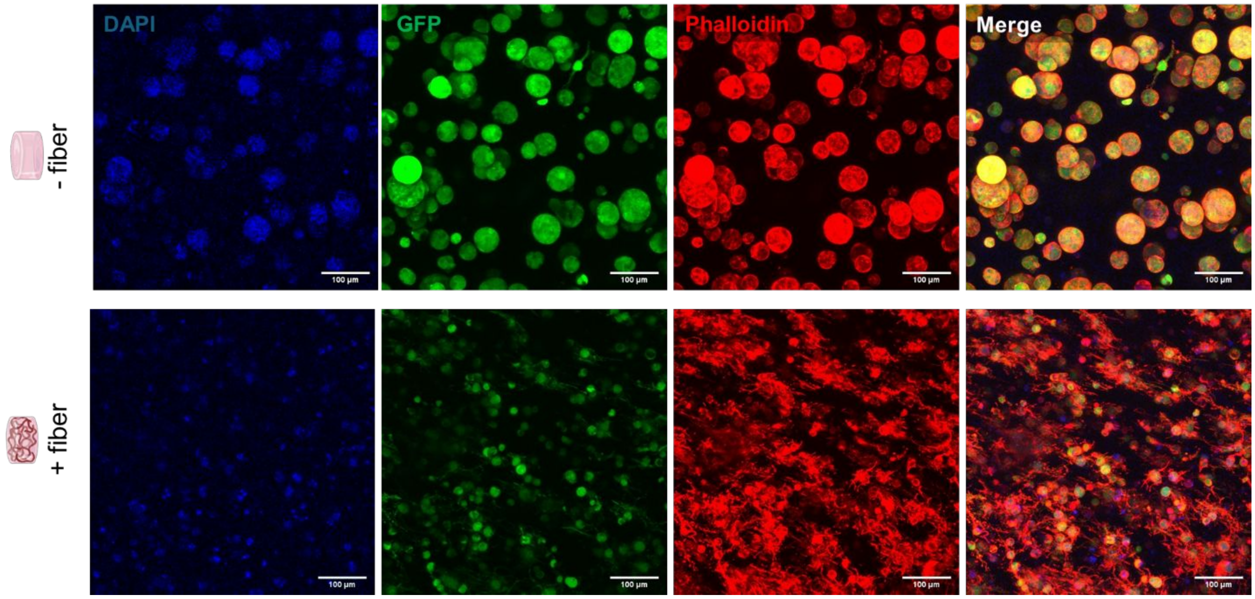

Figure S4. OPCs in – fiber (top row) and + fiber (bottom row) NorHA gels at culture day 7. Individual images are shown for each of the DAPI (blue), GFP (green) and phalloidin (red) channels. DAPI was used to stain cell nuclei, while phalloidin stains for f-actin in the cytoskeleton. The OPC cell line used (GFP+ MADM OPCs) constitutively express GFP in the cytoplasm. Overall, cells in – fiber gels display rounded immature morphologies, while OPCs in + fiber gels extend numerous processes throughout the microtissue.

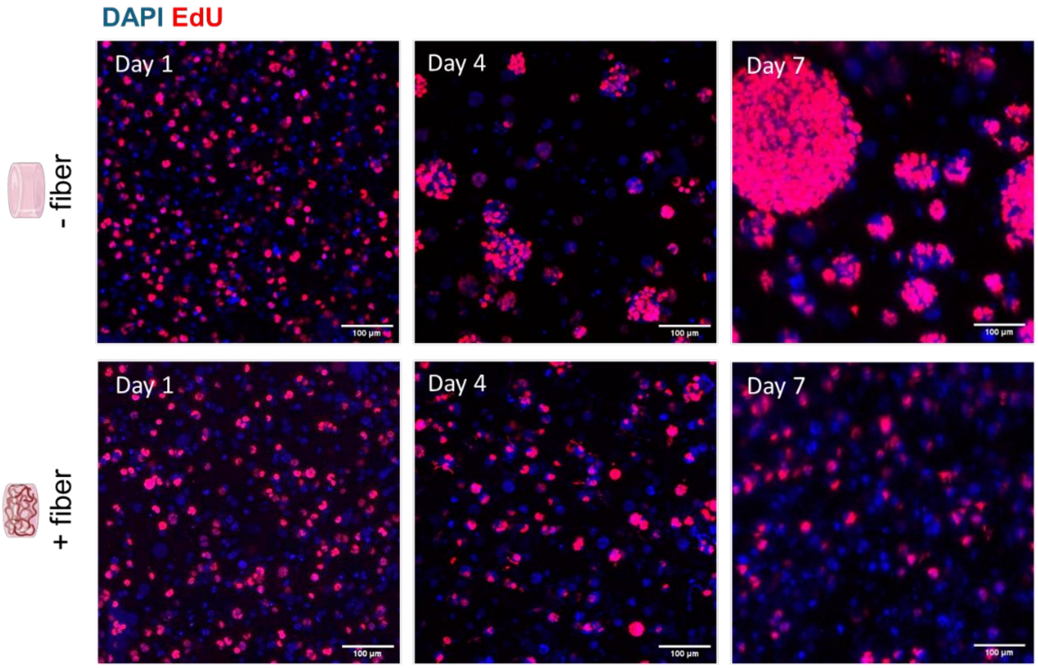

Figure S5. OPCs in – fiber (top row) and + fiber (bottom row) NorHA gels at culture days 1, 4 and 7. DAPI (cell nuclei stain) is labeled in blue while EdU (cell proliferation stain) is labeled in red. Proliferation rate appears similar across both – fiber and + fiber conditions at Day 1. Clusters of highly proliferative cells can be seen in – fiber gels at day 4, with large clusters in evidence by day 7. By contrast, no evident clusters are present in + fiber gels, and proliferation remains consistent between days 1 and 4, with a slight decline by day 7.

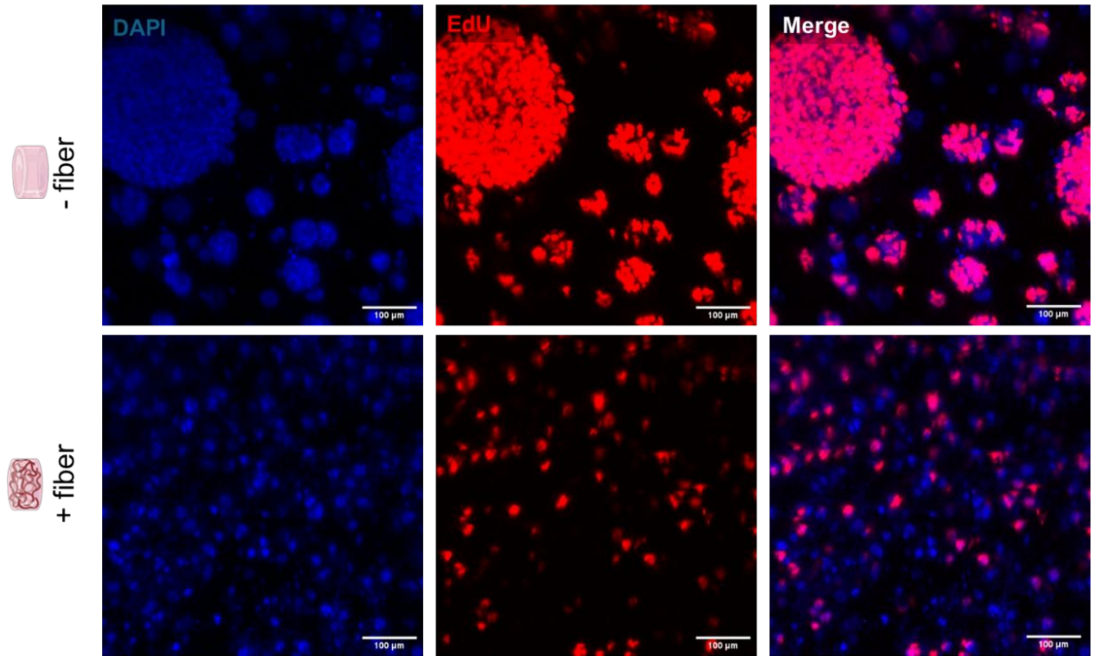

Figure S6. OPCs in – fiber (top row) and + fiber (bottom row) NorHA gels at culture day 7. Individual images are shown for each of the DAPI (blue) and EdU (red) channels. DAPI was used to stain cell nuclei, while EdU labels cells which are actively proliferating. OPCs in the – fiber condition show higher rates of proliferation, particularly within large cell clusters. Proliferation continues to occur in + fiber gels, but to a lesser extent.

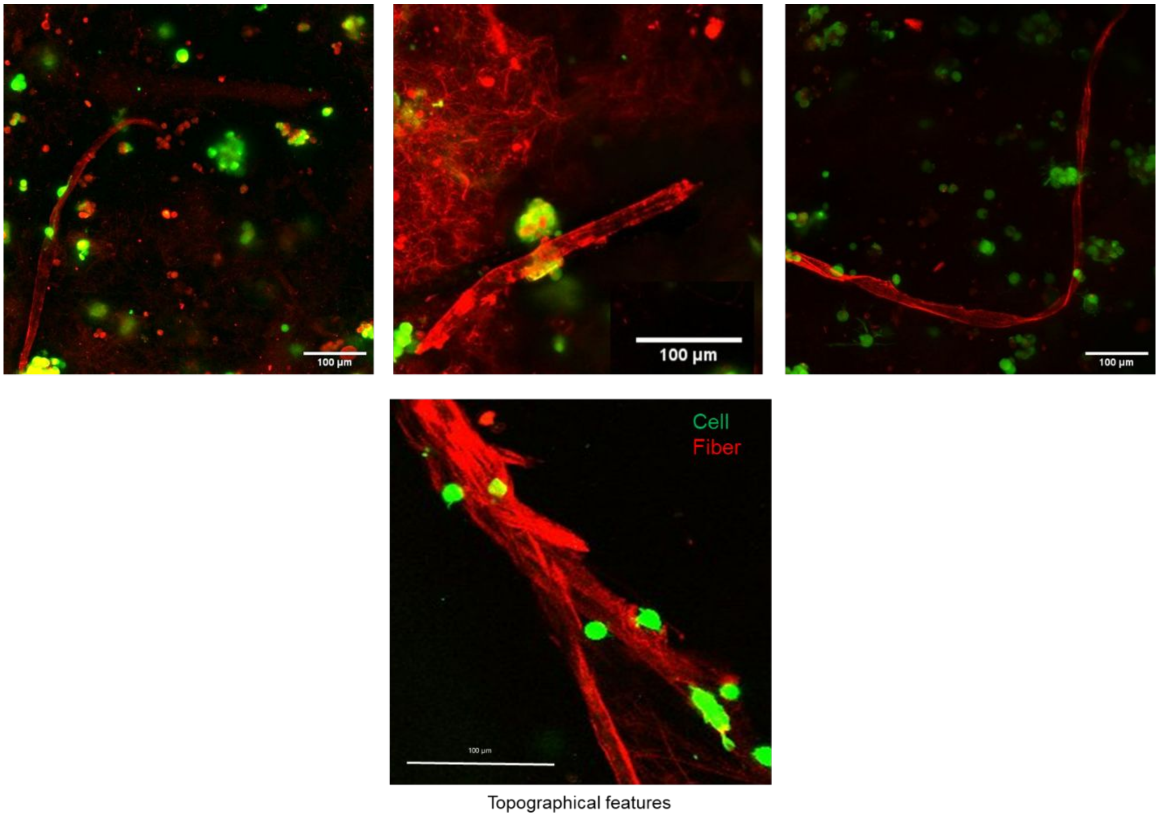

Figure S7. Maximum intensity projections of OPCs (green) grown in proximity to swollen electrospun MeHA fibers (red) in 3D NorHA gels. OPCs are shown to be viable when co-encapsulated with fibers, and in some instances appear to associate with fibers and elongate with fiber direction.

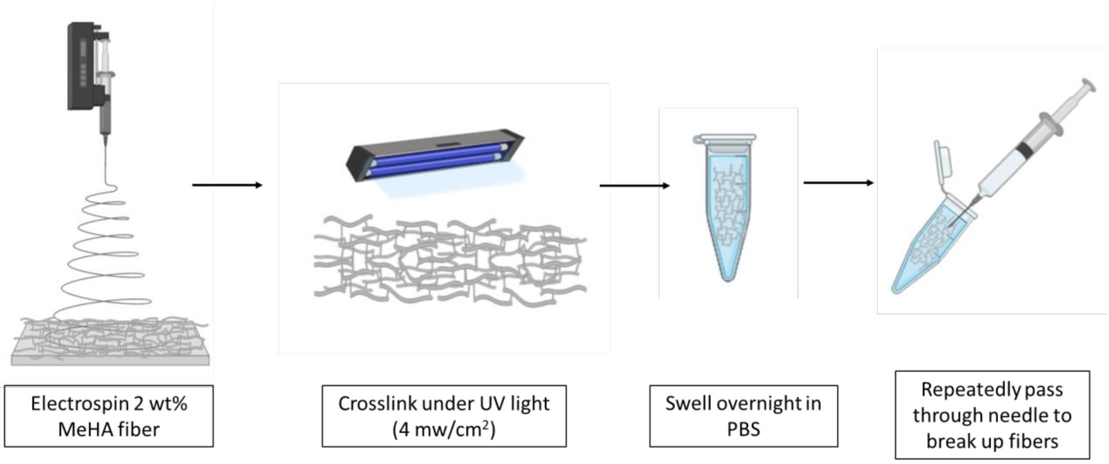

Figure S8. Schematic of fiber processing. Fibers were electrospun followed by crosslinking under UV light. Swelling was conducted in PBS for 24-48 hours until equilibrium was reached. Fiber mats were broken up by repeatedly (20-30x) pushing fiber solution through first an 18G, then a 21G needle.

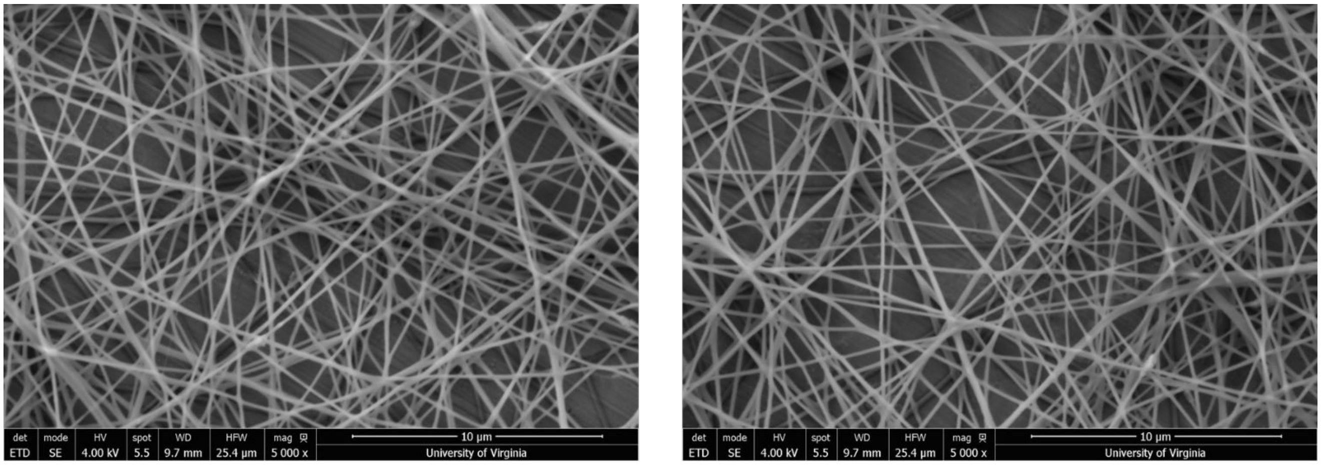

Figure S9. Representative SEM micrographs of dry electrospun MeHA fibers. Fibers exhibit smooth even morphologies with consistent diameters.
